# Cognitive Deficits in Schizophrenia and Other Neuropsychiatric Disorders: Convergence of Preclinical and Clinical Evidence

**DOI:** 10.3389/fnbeh.2014.00444

**Published:** 2014-12-23

**Authors:** Ales Stuchlik, Tomiki Sumiyoshi

**Affiliations:** ^1^Institute of Physiology Academy of Sciences of the Czech Republic, Prague, Czech Republic; ^2^National Center of Neurology and Psychiatry, National Center Hospital, Tokyo, Japan

**Keywords:** cognition, neuropsychiatric disorders, animal models, clinical studies, preclinical studies

Neuropsychiatric disorders, such as schizophrenia, mood disorders, and dementias, produce huge medicinal and socioeconomic burdens for patients and society, posing significant challenges to clinical and preclinical researchers. For example, animal models of neuropsychiatric disorders allow more advanced pharmacological, biochemical, immune-histochemical, electrophysiological, and other techniques to be applied compared to cases with human subjects. Therefore, preclinical investigations are expected to provide valuable information for the development of novel therapeutic options that may benefit patients with specific disorders.

Disturbances of cognitive function, e.g., several types of memory, executive function, attention/information processing, and fluency, are core symptoms of schizophrenia, Alzheimer’s disease (AD), obsessive-compulsive disorder (OCD), epilepsy, etc. In fact, impaired cognition has been shown to negatively affect daily function, sociability, and long-term outcome of patients.

The aim of this e-book is to provide cutting-edge knowledge and reviews of cognitive deficits of neuropsychiatric diseases, in relation to brain function, from a variety of standpoints.

Three intriguing papers (Zawadzki et al., 2013; Fajnerová et al., 2014; Ledoux et al., 2014) report preclinical and clinical evidence for brain correlates of cognitive deficits in schizophrenia. An interesting hypothesis was presented by Nekovarova et al. (2014a) who proposed a translation of findings from animal studies into clinical symptoms of schizophrenia. Specifically, Higuchi et al. described electrophysiological and neuropsychological evidence for cognitive disruptions in subjects at-risk for schizophrenia (Sumiyoshi et al., 2013b; Higuchi et al., 2014). Furthermore, Sumiyoshi et al. (2013a) have provided a theory of a neural basis for atypical antipsychotic drugs to improve cognition in schizophrenia.

Several lines of preclinical evidence on cognitive disturbances are presented. Takashi et al. (2014) found the ability of tandospirone, a 5-HT1A partial agonist, to alleviate aberrant lactate production. Their observations point to a potentially novel therapeutic target for treating schizophrenia-related cognitive deficits. Pei et al. (2014) report divergent phenotypes of neuregulin-1-mutant mice, an animal model of schizophrenia, and the effect of valproate, a mood stabilizer, to improve cognition. Kubík et al. (2014) employed a pharmacological animal model of acute psychosis using the NMDA receptor antagonist MK-801. They found a selective deficit in the co-ordination of multiple informational streams and contextual specificity of neuronal activity, measured by the expression of immediate-early genes.

A totally different serotonergic model of schizophrenia is presented by Rambousek et al. (2014). The study shows effects of psilocin, an active serotonergic hallucinogen of *Psylocibe* mushrooms, on the acquisition, retrieval, and consolidation of memory in two spatial navigation tasks. Another three studies (Kristofikova et al., 2013; Enkel et al., 2014; Petrasek et al., 2014) examined a very promising model of schizophrenia based on transgenic rats with reduced activity of Nogo-A (a protein inhibiting axonal growth). These rats exert neurodevelopmental abnormalities, and disruptions were found in their brain biochemistry, motivation, higher cognitive functions, and circadian rhythms.

Another part was dedicated to AD. Wesierska et al. (2013) show that memantine, a compound used to treat the early stages of AD, improves working memory in a spatial memory task; interestingly only after acute application. The next three papers are clinical, Vlček and Laczó (2014) review neural correlates and spatial orientation changes in mild cognitive impairment and AD. Urbanova et al. (2014) demonstrate an intriguing potential of neurosonology as a non-invasive approach for detecting cerebrovascular disruptions associated with AD, and Marciniak et al. (2014) review beneficial changes in meditation on the cognitive functions associated with aging and neurodegeneration.

Another section is dedicated to depression, which is also accompanied by subtle cognitive deficits, although more pronounced disruptions are seen in the mood and motivation. A theory paper by Nekovarova et al. (2014b) discusses the relation between depression and pain and raises an interesting question, whether antidepressants may also act as analgesics. Holubova et al. (2014) report an antidepressant effect of pregnanolone glutamate, a newly patented steroid derivative previously shown to exert neuroprotective activity and acting mainly via use-dependent inhibition of NMDA receptors. The final part of the book deals with OCD, epilepsy, and three cognition- and neuropsychiatric disorder-related reviews. A report by Hatalova et al. (2014) uses an animal model of OCD based on sensitization by quinpirole, a D2-like receptor agonist, and suggests evidence for disrupted cognitive flexibility tested in spatial memory, cognitive co-ordination, and flexibility tasks. Evidence of cognitive flexibility in OCD in clinical studies yielded mixed results; however, this task (called active place avoidance with reversal on an apparatus called a Carousel) contains a time limitation (animals have to respond within 1 min). Alterations in cognitive flexibility in this model are seen even in an undrugged state in sensitized animals, strongly corroborating the face validity of this particular model of OCD, especially for cognition studies. A relatively novel phenomenon is Internet addiction, which is the topic of another article by Zhou et al. (2013), who examine error-related negativity potentials in Internet-addicted subjects. Two preclinical papers from the field of epileptology (both by Mikulecká et al., 2014a,b) report delayed negative consequences of the postnatal administration of clonazepam on cognitive and social behaviors. This has great significance, since in some countries, clonazepam is used for treating pediatric epileptic patients. These papers suggest that great caution should be used in these prescriptions. Finally, three reviews provide an update for cognition, behavior, and neuropsychiatric disorders; Stuchlik (2014) discusses memory in dynamic environments and its relation to synaptic plasticity. Takeuchi et al. (2014) examined, for the first time, the effect of sleep on the organization of memory processes. Finally, Karatsoreos (2014) reviews relationships between circadian rhythms and neuropsychiatric disorders.

The Book provides an up-to-date review on the integration of preclinical and clinical approaches to cognitive deficits in neuropsychiatric disorders. Our edition is expected to give greater insight into treatment options with higher benefit/risk ratios.

## Conflict of Interest Statement

The authors declare that the research was conducted in the absence of any commercial or financial relationships that could be construed as a potential conflict of interest.
